# Ketamine Induces Apoptosis and Inhibits Proliferation in HT-29 Colorectal Cancer Cells

**DOI:** 10.3390/biomedicines14040907

**Published:** 2026-04-16

**Authors:** Irmak Fatoş Korkmaz, Tugba Elgun, Çiğdem Aktas, Ersin Gündeğer, Asiye Gok Yurttas

**Affiliations:** 1 Department of Anaesthesia, University of Health Science, Bahçelievler State Hospital, Istanbul 34668, Türkiye; korkmazfatos1112@gmail.com; 2 Medical Biology, Faculty of Medicine, Biruni University, Istanbul 34015, Türkiye; telgun@biruni.edu.tr; 3 Biruni University Research Center (B@MER), Biruni University, Istanbul 34015, Türkiye; 4 Department of Genetics, Aziz Sancar Institute, Istanbul University, Istanbul 34452, Türkiye; cigdemaktas485@gmail.com; 5 Department of Biochemistry, Faculty of Pharmacy, Istanbul Health and Technology University, Istanbul 34275, Türkiye; ersin.gundeger@istun.edu.tr; 6 Department of Medical Biochemistry, Faculty of Medicine, Istanbul Atlas University, Istanbul 34408, Türkiye

**Keywords:** ketamine, colorectal cancer cells, cell viability, carcinoma, apoptosis

## Abstract

**Background**: Colorectal cancer (CRC) is one of the most prevalent malignancies worldwide and remains a major health concern due to its high recurrence and mortality rates. Recent studies suggest that anesthetic agents, including ketamine, may have direct effects on cancer cell viability and apoptosis. Objective: This study aimed to investigate the in vitro effects of ketamine on the HT-29 human colorectal adenocarcinoma cell line, focusing on its cytotoxic and pro-apoptotic potential. **Material and Methods**: HT-29 cells were treated with ketamine for 24 h. Cell viability was evaluated using the MTT assay. Apoptosis rates were determined by flow cytometry with Annexin V-FITC/7-AAD staining. Furthermore, quantitative PCR (qPCR) was performed to assess the expression levels of key genes associated with proliferation and apoptosis. GeneQuery™ Human Basal Cell Carcinoma qPCR Array Kit (GQH-BCC-GK015-C) was used for qPCR analysıs. Molecular docking simulations were performed to investigate the potential molecular interactions between ketamine and three target proteins: the N-methyl-D-aspartate (NMDA) receptor, epidermal growth factor receptor (EGFR), and casein kinase 1 delta (CSNK1D). To ensure robustness of predictions, two independent docking methods were employed. **Results**: Ketamine significantly reduced cell viability in a dose-dependent manner, with an IC_50_ value of approximately 1.05 µM. Flow cytometry analysis demonstrated a marked increase in early apoptosis (23.9%) in treated cells. These findings suggest that ketamine exhibits potential anti-proliferative and pro-apoptotic effects on HT-29 colorectal cancer cells. **Conclusions**: These findings suggest that ketamine exhibits potential anti-proliferative and pro-apoptotic effects on HT-29 colorectal cancer cells in vitro. Further studies are warranted to elucidate the underlying molecular mechanisms and potential clinical implications.

## 1. Introduction

Colorectal cancer (CRC) ranks among the leading malignancies worldwide and continues to account for a considerable proportion of cancer-related deaths [1]. Although significant progress has been achieved through surgical procedures and chemotherapeutic strategies, recurrence and metastatic spread still pose critical obstacles to successful treatment [2,3,4]. In parallel with these clinical challenges, researchers have begun to explore how anesthetic drugs might influence tumor cell dynamics during the perioperative period [5]

Ketamine, widely recognized as an NMDA receptor antagonist used in anesthesia, has been shown to exert biological effects beyond its anesthetic role. Recent experimental data indicate that this compound can directly impair cancer cell proliferation and promote apoptotic mechanisms in various tumor models, including gastric, lung, and breast cancers [6,7,8,9]. However, its specific impact on colorectal cancer cells, particularly the HT-29 line, remains insufficiently characterized.

The primary objective of this study was to examine the impact of ketamine on HT-29 colorectal adenocarcinoma cells in vitro. Specifically, we evaluated its influence on cell survival, induction of apoptosis, and the regulation of apoptosis-related genes. Understanding these effects is important, since accumulating evidence indicates that anesthetic drugs can affect cancer cell behavior by interfering with proliferation and cell death pathways [5,10]. For example, ketamine has been shown to modulate tumor growth in gastric and lung cancer models through alterations in the PI3K/AKT and caspase signaling cascades [6]. However, the available data on colorectal cancer remain limited, particularly in relation to the HT-29 cell line. By addressing this gap, our research aims to provide further insights into whether ketamine, beyond its anesthetic role, could also contribute to modulating cancer progression during and after surgery [7].

Colorectal cancer progression is regulated by multiple interconnected signaling pathways, including Wnt/β-catenin, EGFR, and inflammatory signaling networks. Recent studies have highlighted the importance of targeting these pathways to inhibit tumor growth and metastasis [11]. In addition, emerging evidence suggests that drug repurposing strategies, including the use of anesthetic agents, may provide novel therapeutic opportunities in colorectal cancer management [12,13,14].

## 2. Material and Methods

### 2.1. Cell Culture

The human colorectal adenocarcinoma cell line HT-29 was obtained from the American Type Culture Collection (ATCC, Manassas, VA, USA). Cells were maintained in DMEM/F-12 medium supplemented with 10% fetal bovine serum (FBS, Gibco, Grand Island, NY, USA) and 1% penicillin–streptomycin (100 U/mL, Gibco, Grand Island, NY, USA). Cultures were incubated at 37 °C in a humidified atmosphere containing 5% CO_2_. When cells reached 80–90% confluency, subculturing was performed using 0.25% trypsin–EDTA (Gibco, Grand Island, NY, USA). To ensure phenotypic stability, experiments were conducted with cells between passages 5 and 15.

### 2.2. Ketamine Treatment

Ketamine (racemic ketamine, RS-ketamine; Sigma-Aldrich, St. Louis, MO, USA) was used in this study. A stock solution was prepared in sterile distilled water and subsequently diluted in culture medium to final concentrations of 0.5, 1, 2.5, and 5 μM.

### 2.3. MTT Assay

Cell viability was evaluated using the MTT [3-(4,5-dimethylthiazol-2-yl)-2,5-diphenyltetrazolium bromide] assay. HT-29 cells were seeded into 96-well culture plates at a density of 1 × 10^4^ cells per well and allowed to attach for 24 h. Subsequently, the cells were exposed to the specified concentrations of ketamine for 24 h. After the treatment period, 100 μL of MTT solution (0.4 mg/mL; Sigma-Aldrich St. Louis, MO, USA,) was added to each well, followed by a 3 h incubation at 37 °C. The medium was carefully removed, and 100 μL of dimethyl sulfoxide (DMSO, Merck, Darmstadt, Germany) was added to solubilize the formazan crystals. The optical density was subsequently determined at 550 nm using a microplate reader (BioTek Instruments, Winooski, VT, USA). All experimental groups were analyzed in triplicate across three independent biological replicates.

### 2.4. Apoptosis Analysis

Apoptosis was evaluated using the Annexin V-FITC/7-AAD apoptosis detection kit (BioLegend, San Diego, CA, USA). HT-29 cells were seeded in 6-well plates at a density of 6 × 10^5^ cells per well and maintained for 24 h. Subsequently, the cells were exposed to 1 μM ketamine for an additional 24 h. After treatment, the cells were collected using trypsinization, washed twice with PBS, and resuspended in binding buffer. Annexin V-FITC and 7-AAD staining were then carried out in accordance with the manufacturer’s protocol. Fluorescence signals were acquired using a BD Accuri C6 Plus flow cytometer (BD Biosciences, Franklin Lakes, NJ, USA), and data were analyzed to quantify viable, early apoptotic, late apoptotic, and necrotic populations. The findings were derived from a minimum of three independent experimental replicates.

### 2.5. RNA Isolation and Quantitative Real-Time PCR

Total RNA was isolated from HT-29 cells using the innuPREP RNA Mini Kit 2.0 (Analytik Jena, Jena, Germany) following the manufacturer’s instructions. The concentration and purity of the extracted RNA were determined with a NanoDrop 2000 spectrophotometer (Thermo Fisher Scientific, Wilmington, DE, USA). Subsequently, 1 μg of RNA was reverse transcribed into complementary DNA (cDNA) utilizing the OneScript^®^ Plus cDNA Synthesis Kit (Applied Biological Materials Inc. [ABM], Richmond, BC, Canada). Quantitative PCR (qPCR) analysis was performed on the Roche LightCycler^®^ 480 platform with the GeneQuery Human Basal Cell Carcinoma Array Kit (ScienCell Research Laboratories, GQH-BCC-GK015-C, Carlsbad, CA, USA). Each reaction was prepared in a final volume of 20 μL containing 10 μL of 2× GoldNStart TaqGreen Master Mix (Applied Biological Materials Inc. [ABM], Richmond, BC, Canada) and conducted under the following thermal profile: an initial denaturation at 95 °C for 10 min, followed by 40 amplification cycles of 95 °C for 20 s, 65 °C for 20 s, and 72 °C for 20 s. All samples were analyzed in duplicate. LDHA served as the internal reference gene, and relative gene expression was quantified using the 2^−ΔΔCq^ method. LDHA was selected as the reference gene due to its stable expression across experimental conditions, as recommended by the manufacturer. The qPCR array used includes predefined primer sets, and therefore individual primer sequences are not provided separately.

Although the GeneQuery™ Human Basal Cell Carcinoma qPCR Array Kit is not specifically designed for colorectal cancer, it includes key genes involved in fundamental oncogenic pathways such as Wnt/β-catenin and EGFR signaling. These pathways are highly relevant to colorectal cancer biology; therefore, the panel was used to explore general cancer-related molecular responses. However, this represents a limitation of the study.

### 2.6. Molecular Docking Analysis

Molecular docking simulations were performed to investigate the potential molecular interactions between ketamine and three target proteins: the N-methyl-D-aspartate (NMDA) receptor, epidermal growth factor receptor (EGFR), and casein kinase 1 delta (CSNK1D). To ensure robustness of predictions, two independent docking methods were employed: AutoDock Vina [15,16,17].

Protein preparation: The three-dimensional crystal structures of the NMDA receptor (PDB ID: 7EU8, resolution 2.90 Å) [18] (EGFR (PDB ID: 4HJO, resolution 2.40 Å) [19] and CSNK1D (PDB ID: 4TW9, resolution 2.00 Å) [20] were retrieved from the RCSB Protein Data Bank. Water molecules, ions, and co-crystallized ligands were removed during preparation. For AutoDock Vina, protein preparation initially attempted protonation state assignment at pH 7.4 using PDB2PQR with PROPKA3 [21]; however, due to structural incompatibilities with input PDB files, polar hydrogen atoms were ultimately assigned using OpenBabel [22] (obabel -xp -h flags). For Schrödinger Glide, protein structures were prepared using the Protein Preparation Wizard [23], which included hydrogen addition, protonation state assignment at pH 7.4, and energy minimization using the OPLS4 force field [24].

Ligand preparation: The three-dimensional structure of ketamine was generated from its canonical SMILES representation (CNC1(CCCCC1=O)C1=CC=CC=C1Cl). For AutoDock Vina, conformational sampling was performed using the ETKDGv3 distance geometry algorithm [25] implemented in RDKit (version 2025.09.3), generating 50 conformers with randomSeed set to 42 for reproducibility. Each conformer was subsequently energy-minimized using the Merck Molecular Force Field (MMFF) [26] with default parameters (maximum 200 iterations). The lowest-energy conformer was converted to PDBQT format using Meeko [27] for docking. For Schrödinger Glide, ligand preparation was conducted using LigPrep [28], which generated ionization states at pH 7.4 ± 2.0 and produced tautomeric forms. All conformers were energy-minimized using the OPLS4 force field [24].

Docking protocols: For AutoDock Vina 1.2.3 [16], the docking search space was defined as a cubic grid box of 22 × 22 × 22 Å^3^ centered on the geometric center of co-crystallized ligands within each target protein’s active site. The exhaustiveness parameter was set to 16 to ensure adequate conformational sampling, and 20 binding modes were generated per target. For Schrödinger Glide, molecular docking was performed using the Standard Precision (SP) mode [29] with default parameters. The receptor grid was generated using a 22 Å cubic box centered on co-crystallized ligand positions. Both protocols were validated by redocking co-crystallized ligands (JC9 for NMDA, AQ4 for EGFR, 386 for CSNK1D) into their respective binding sites [30,31].

Analysis: Binding affinities from AutoDock Vina are reported as mean ± standard deviation across all docked conformers and binding modes. Schrödinger Glide docking scores represent the best-scoring pose for each target. Two-dimensional ligand-protein interaction diagrams were generated using the Schrödinger Ligand Interaction Diagram tool to identify key residues involved in binding, including hydrogen bonds, salt bridges, and hydrophobic contacts.

### 2.7. Statistical Analysis

Statistical analyses were performed using (GraphPad Prism software, version 9.1; San Diego, CA, USA). Data are presented as mean ± standard deviation (SD) from at least three independent experiments (*n* = 3), with each experiment performed in triplicate. In this study, ‘groups’ are defined as the distinct experimental conditions involving cells treated with different concentrations of ketamine (0.5, 1, 2.5, and 5 μM) and the untreated control group. Comparisons between two groups were assessed using Welch’s corrected unpaired *t*-test, while differences among multiple groups were evaluated by one-way ANOVA followed by Tukey–Kramer post hoc analysis. A *p*-value of less than 0.05 was considered indicative of statistical significance.

## 3. Results

### 3.1. Ketamine Reduces HT-29 Cell Viability in a Dose-Dependent Manner

**Figure 1:**
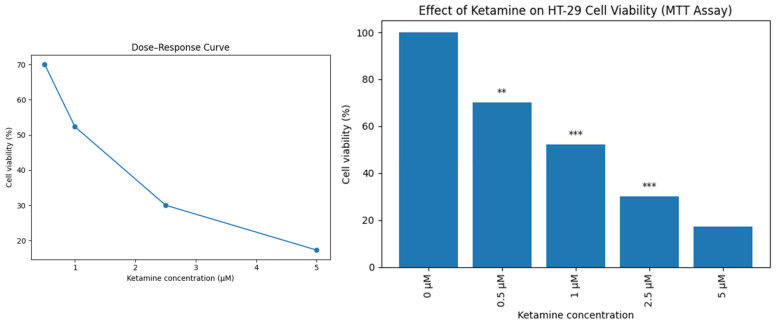
The cell viabilities (%) of HT-29 cells were calculated according to the results of the MTT assay (One-way ANOVA test, ** *p* < 0.01; *** *p* < 0.001).

A pronounced decline in the viability of HT-29 colorectal adenocarcinoma cells was observed following ketamine treatment. According to the MTT assay, the mean viability progressively declined from 100% in untreated controls to 70.06%, 52.32%, 30.03%, and 17.29% at concentrations of 0.5, 1, 2.5, and 5 μM, respectively. Statistical evaluation by one-way ANOVA demonstrated highly significant differences among groups (F = 98.96, *p* < 0.001). Tukey’s post hoc analysis further confirmed that all ketamine-treated groups exhibited significantly reduced viability compared with the control group (*p* < 0.01 at 0.5 μM; *p* < 0.001 at ≥1 μM). The calculated IC_50_ value was approximately 1.05 μM, indicating potent cytotoxic activity of ketamine at low micromolar concentrations (Figure 1).

### 3.2. Induction of Apoptosis by Ketamine

**Figure 2:**
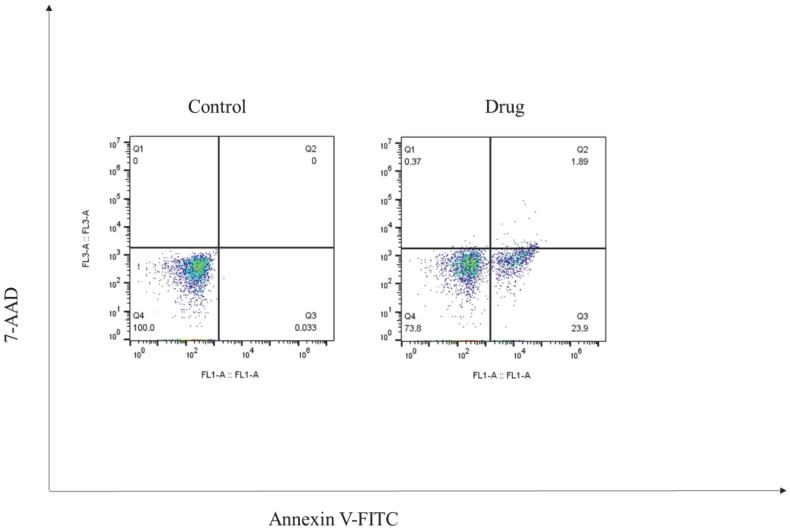
Detection of apoptosis in HT-29 cells using Annexin V/7-AAD staining.

The impact of ketamine on apoptotic cell death was evaluated by flow cytometry following Annexin V-FITC/7-AAD staining. After 24 h of treatment with 1 μM ketamine, a notable increase in the proportion of apoptotic cells was detected. In particular, the percentage of early apoptotic cells reached 23.9%, whereas only minimal levels were observed in the untreated control group. Late apoptotic cells accounted for 1.89%, while necrotic cells remained low (0.37%). These results suggest that ketamine primarily induces programmed cell death via early apoptosis rather than necrosis (Figure 2).

### 3.3. Alterations in Gene Expression

Twenty-nine target genes were evaluated in HT-29 cells after viability testing. Gene expression was quantified using the 2^−ΔΔCq^ approach, followed by log2 transformation to normalize fold-change distribution. This adjustment ensured that increases did not scale disproportionately while reductions remained interpretable. Expression profiling emphasized genes relevant to oncogenic signaling (Table 1). qPCR analysis revealed marked downregulation of EGF, TCF7, and CSNK1D, meeting the established threshold for HT-29 cells. Gene expression data were analyzed using two-way ANOVA, and Duncan’s post hoc procedure was applied to identify significant group differences. Results meeting the criterion of *p* < 0.001 were reported. Expression values above 2, based on the 2^−ΔΔCq^ method, were taken to indicate gene upregulation, while values below 0.5 reflected downregulation.

### 3.4. Dual-Method Molecular Docking Validates NMDA Receptor as Primary Ketamine Target

Molecular docking was performed using two independent methods (AutoDock Vina and Schrödinger Glide) to evaluate the binding potential of ketamine toward three protein targets [32,33]. The comparative results are summarized in Table 2.

Both docking methods consistently identified the NMDA receptor as the strongest ketamine binding target. AutoDock Vina yielded a best binding energy of −6.79 kcal/mol (mean: −6.02 ± 0.32 kcal/mol), while Schrödinger Glide produced a docking score of −7.53 kcal/mol, demonstrating strong concordance between methods. In contrast, both approaches predicted substantially weaker interactions with EGFR and CSNK1D, with AutoDock Vina scores of −6.00 and −5.90 kcal/mol, respectively, and Schrödinger Glide scores of −4.43 and −5.96 kcal/mol. AutoDock Vina redocking of co-crystallized ligands yielded RMSD values of 4.12–9.50 Å, with higher values for EGFR reflecting the substantial conformational flexibility of this receptor tyrosine kinase [30,31].

**Figure 3:**
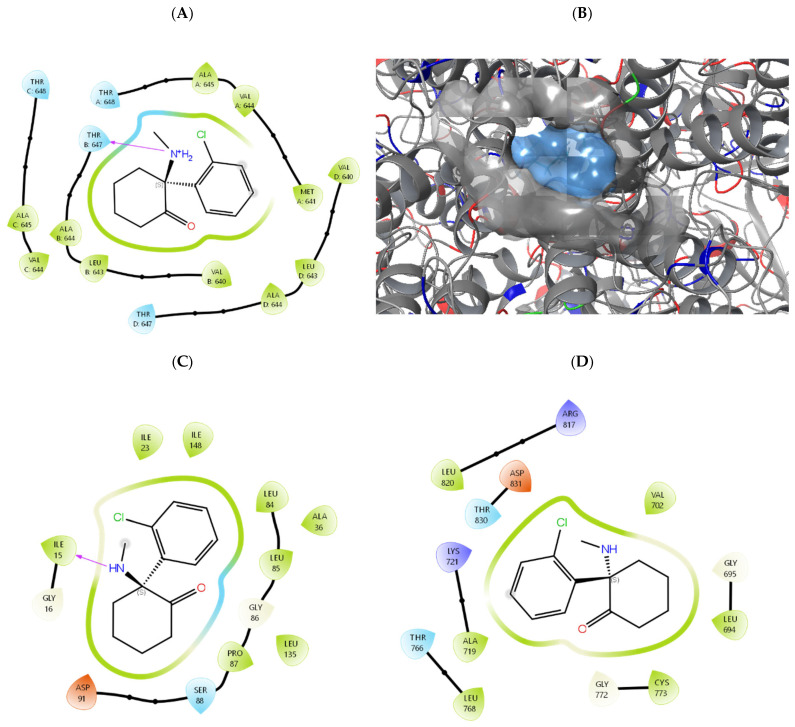
Hydrogen bonds are shown as dashed lines, and hydrophobic interactions are indicated by green residues. Ketamine binding modes and interaction profiles. (**A**) Predicted binding poses for ketamine in the NMDA receptor channel pore. (**B**) Bottom-up visualization of three-dimensional ligand interaction diagrams in ketamine binding to NMDA receptor. (**C**) CSNK1D and (**D**) EGFR. Hydrophobic residues are shown in green, polar residues in cyan, charged residues in orange/blue. Hydrogen bonds are depicted as purple arrows.

Detailed interaction analysis revealed that ketamine occupies a predominantly hydrophobic channel within the NMDA receptor pore, forming critical contacts with residues Ala644, Val640, Leu643, and Thr647/648 across multiple subunits (Figure 3). A key hydrogen bond was observed between the protonated amine group of ketamine and Thr647 in the B subunit. In contrast, ketamine binding to EGFR and CSNK1D involved fewer specific interactions and was characterized by lower predicted affinities across both docking methods.

## 4. Discussion

This study provides evidence that ketamine elicits strong, dose-dependent cytotoxic responses in HT-29 colorectal cancer cells cultured in vitro. The observed decrease in cell viability, verified through ANOVA with Tukey’s post hoc test, is consistent with earlier findings indicating that ketamine inhibits cellular proliferation and promotes apoptotic pathways in different cancer models. The calculated IC_50_ value of approximately 1.05 µM indicates that ketamine is effective at relatively low concentrations. These findings support the hypothesis that ketamine, beyond its anesthetic role, may have potential as an adjuvant agent in oncological surgery by directly affecting tumor cell survival. Nevertheless, additional comprehensive investigations, incorporating apoptosis assays and gene expression analyses, are required to clarify the molecular mechanisms involved and to assess the potential clinical significance.

Recent evidence indicates that ketamine may suppress tumor cell growth by influencing apoptotic pathways. For example, [6] demonstrated that ketamine induced apoptosis in lung carcinoma cells through the upregulation of caspase-3 and downregulation of Bcl-2 expression. In colorectal cancer models, however, the effects of ketamine remain less well characterized. Our findings extend this evidence to HT-29 cells, suggesting that ketamine may exhibit similar pro-apoptotic and anti-proliferative effects in colorectal cancer.

The observed IC_50_ value (~1.05 µM) is comparable to or lower than those reported for several anti-cancer agents in colorectal cancer cell models, suggesting that ketamine may exert biologically relevant effects at relatively low concentrations [34]. The significant reduction in cell viability at concentrations as low as 0.5 µM further supports the notion that ketamine’s cytotoxicity is dose-dependent.

It is increasingly recognized that perioperative factors, including anesthetic choice, can influence cancer outcomes [10]. While the primary role of ketamine is as an anesthetic and analgesic agent, these findings raise the possibility that it could contribute to tumor suppression during surgical management of colorectal cancer. However, this potential must be interpreted cautiously. As this study was performed in vitro, the cytotoxic effects observed may not entirely reflect the responses within the complex in vivo tumor microenvironment.

Further studies are needed to elucidate the precise molecular mechanisms underlying ketamine’s impact on CRC cells, including detailed analysis of apoptosis markers and signaling pathways such as PI3K/AKT, NF-κB, and caspase cascades. Moreover, combining ketamine with conventional chemotherapeutic agents could be explored as a strategy to enhance anti-tumor efficacy, as suggested by studies in other tumor types [35].

The present study revealed that ketamine promotes substantial apoptosis in HT-29 colorectal cancer cells in vitro, as indicated by flow cytometric analysis. The observed increase in early apoptosis (23.9%) suggests that ketamine effectively triggers programmed cell death pathways shortly after exposure. The relatively low percentage of late apoptotic cells (1.89%) and negligible necrosis (0.37%) indicate that the primary effect of ketamine is to initiate early-stage apoptosis without causing extensive membrane damage or uncontrolled cell lysis.

These results are consistent with previous reports highlighting the pro-apoptotic properties of ketamine in various cancer cell lines. For example, [8] demonstrated that ketamine induces apoptosis in human lung carcinoma cells through mitochondrial pathways involving Bax and caspase-3 upregulation and Bcl-2 downregulation. Similar apoptotic effects have been reported in breast and gastric cancer cells, supporting the notion that ketamine may modulate key molecular regulators of apoptosis [2].

In this study, ketamine exposure led to a marked downregulation of EGF, TCF7, and CSNK1D expression in HT-29 colorectal adenocarcinoma cells (log_2_FC ≈ 0.5). These results indicate that ketamine may suppress multiple signaling pathways critical for colorectal cancer cell proliferation, migration, and invasion.

Epidermal Growth Factor (EGF) is a critical ligand that activates the MAPK/ERK and PI3K/AKT signaling cascades via binding to its receptor EGFR. These pathways play central roles in promoting cell growth, survival, and motility (Qu et al., 2025) [36]. Recent studies have demonstrated that EGF signaling enhances MMP-9 expression through ERK1/2 and AKT phosphorylation, as well as downstream activation of NF-κB, thereby facilitating tumor cell invasiveness [37,38]. The observed downregulation of EGF following ketamine treatment may lead to suppression of these cascades, resulting in decreased MMP-9 mediated extracellular matrix degradation and reduced migratory capacity.

TCF7, a key nuclear transcription factor of the Wnt/β-catenin pathway, is frequently upregulated in colorectal cancer and regulates the expression of target genes involved in tumor progression [39]. Hyperactivation of Wnt signaling induces expression of metastasis-associated genes including MMP-9 [40] (Zhao et al., 2022). The downregulation of TCF7 by ketamine is likely to reduce Wnt signaling activity, which may in turn suppress the invasive and metastatic capacity of colorectal cancer cells.

Casein Kinase 1 Delta (CSNK1D) is a serine/threonine kinase that modulates multiple signaling pathways. Recent evidence supports CSNK1D’s role in stabilizing β-catenin and promoting colorectal tumor progression via Wnt signaling [41,42]. Moreover, CSNK1D has been shown to crosstalk with AKT and ERK pathways to facilitate cell proliferation and survival [43]. The suppression of CSNK1D expression by ketamine disrupts these coordinated signaling networks, leading to attenuation of malignant phenotypes.

In summary, ketamine-induced downregulation of EGF, TCF7, and CSNK1D genes suggests a potential association with the modulation of ERK and AKT signaling pathways, which may contribute to decreased MMP-9 expression and reduced proliferative and invasive capacity. However, these findings are based on transcriptional data and do not represent direct evidence of pathway inhibition [35,44].

Taken together, these results add to the accumulating evidence that anesthetic agents, including ketamine, can exert direct antitumor activity. However, further studies are warranted to elucidate the underlying molecular mechanisms and to investigate whether these in vitro effects translate to clinically meaningful outcomes during perioperative management of colorectal cancer patients [35,45].

It should also be considered that ketamine exists in different enantiomeric forms, including S-ketamine, R-ketamine, and the racemic mixture (RS-ketamine), each exhibiting distinct pharmacological properties [18,46]. In the present study, racemic ketamine was used, which represents the most commonly employed form in clinical anesthesia. However, S-ketamine has been reported to possess higher affinity for NMDA receptors and greater biological potency compared to the racemic form [18,47]. Therefore, it is plausible that the anti-proliferative and pro-apoptotic effects observed in HT-29 cells could differ in magnitude if enantiomer-specific formulations were used. In particular, S-ketamine may exert more pronounced effects on apoptosis induction, oxidative stress regulation, and downstream signaling pathways such as NMDA receptor–mediated CaMKII and c-Myc signaling [46,48]. These considerations highlight the importance of enantiomer-specific investigations in future studies to better define the molecular and therapeutic implications of ketamine in colorectal cancer models.

The molecular docking analysis, validated through two independent computational methods, provides strong computational support for ketamine’s primary mechanism of action through NMDA receptor antagonism [32,33]. The consensus between AutoDock Vina and Schrödinger Glide—both identifying NMDA receptor as the strongest binding target—significantly strengthens confidence in the computational predictions. This dual-method approach addresses known limitations of individual docking algorithms, which can vary in their scoring functions and conformational sampling strategies [32,33]

The predicted binding affinities for the NMDA receptor (−6.79 and −7.53 kcal/mol) are consistent with ketamine’s known pharmacological profile as an NMDA receptor channel blocker, with reported Ki values of approximately 0.53–1.06 μM [18,46]. The structural basis for ketamine binding to the NMDA receptor channel pore has been confirmed by cryo-EM studies [18], demonstrating that S-ketamine occupies a site overlapping with the memantine binding pocket between GluN1 and GluN2 subunits. Our docking poses closely recapitulate this experimental binding mode, with ketamine positioned within the hydrophobic channel and forming hydrogen bonds with conserved threonine residues that gate the pore [18].

Importantly, both docking methods predicted substantially weaker binding to EGFR (−6.00 vs. −4.43 kcal/mol) and CSNK1D (−5.90 vs. −5.96 kcal/mol), despite both proteins being downregulated in our experimental qPCR analyses (EGF and CSNK1D genes). This computational finding supports the hypothesis that ketamine’s effects on these pathways are mediated indirectly through downstream signaling cascades rather than direct protein inhibition. This interpretation is mechanistically consistent with established NMDA receptor signaling biology: NMDA receptor blockade reduces intracellular calcium influx, which in turn decreases CaMKII phosphorylation and c-Myc transcriptional activity [35,48]. Since c-Myc functions as a master regulator controlling numerous proliferative genes, its downregulation following NMDA receptor antagonism could account for the observed changes in EGF and CSNK1D expression [48].

Furthermore, significant crosstalk exists between NMDA receptor signaling and both the Wnt/β-catenin pathway (in which CSNK1D participates) and EGFR signaling networks. NMDA receptors have been shown to physically associate with EGFR in heterocomplex formations, providing a structural basis for functional interaction between glutamatergic and growth factor signaling. Similarly, CaMKII—a direct downstream target of NMDA receptor activation—participates in an auto-activating loop with Wnt signaling that affects TCF transcription factor expression in colorectal cancer cells. These pathway-level interactions provide a mechanistic framework for understanding how upstream NMDA receptor blockade propagates through interconnected signaling networks to affect EGFR and CSNK1D pathway activity without requiring direct drug-target binding. However, it should be noted that these findings are based solely on mRNA expression levels and were not validated at the protein level. Since transcriptional changes do not always correlate with protein expression or functional activity, further studies using protein-level analyses such as Western blotting or ELISA are required.

The correlation between computational binding predictions and our experimental IC50 of approximately 1.05 μM requires interpretation within the known limitations of docking scoring functions [32,33]. While ketamine’s predicted NMDA receptor affinity corresponds reasonably well with literature Ki values (~0.5–1.0 μM) [18,46], absolute docking scores should not be directly equated with experimental binding constants. Docking algorithms are optimized primarily for pose prediction rather than absolute free energy calculation, with typical scoring function errors of 1.5–3.0 kcal/mol [30,32]. Nevertheless, the consistent ranking of NMDA receptor as the strongest target across two independent methods provides confidence that the computational predictions reflect genuine differences in binding affinity [32,33].

Detailed analysis of the predicted binding modes reveals that ketamine occupies a highly hydrophobic channel within the NMDA receptor pore region, consistent with its function as an uncompetitive (channel-blocking) antagonist [18]. The binding pocket is predominantly lined with hydrophobic residues (Ala644, Val640, Leu643, Ile23, Ile148, Leu84/85) that stabilize ketamine’s chlorophenyl ring and cyclohexanone scaffold through van der Waals contacts. The protonated amine group of ketamine, which is physiologically relevant at pH 7.4 (pKa ~ 7.5), forms a critical hydrogen bond with Thr647 in the GluN2B subunit. This interaction is consistent with experimental mutagenesis data showing that threonine residues at this position are essential for ketamine binding and channel block [18]. The predicted binding orientation positions ketamine within the narrowest region of the channel pore, thereby physically occluding ion permeation—the hallmark of uncompetitive NMDA antagonism [18,46].

In contrast, ketamine binding to EGFR and CSNK1D exhibited fewer specific stabilizing interactions. For EGFR, ketamine was predicted to bind within the ATP-binding pocket but with predominantly nonspecific hydrophobic contacts and weak predicted affinities across both methods. For CSNK1D, binding occurred at a surface-exposed site distinct from the active kinase domain, suggesting limited functional relevance. These computational observations support the conclusion that EGFR and CSNK1D downregulation observed in our study likely reflects indirect transcriptional effects rather than direct enzyme inhibition. While the use of two independent docking methods strengthens our conclusions [32,33], several limitations should be acknowledged. Both methods employed rigid receptor docking, which may not adequately capture induced-fit conformational changes upon ligand binding—particularly relevant for EGFR, which undergoes substantial structural rearrangements during activation [31]. Additionally, empirical scoring functions used by both AutoDock Vina [16] and Schrödinger Glide [17,29] provide approximations of binding free energies that do not explicitly account for entropic contributions or explicit solvation effects [31]. The observed discrepancy in EGFR scores between methods (−6.00 vs. −4.43 kcal/mol) likely reflects differences in scoring function parameterization and conformational sampling, with Schrödinger Glide’s more conservative estimate potentially more reliable for this flexible kinase target.

Redocking validation yielded RMSD values exceeding the conventional 2.0 Å threshold (4.12–9.50 Å), particularly for EGFR (9.50 Å) [30].These elevated values reflect the substantial conformational flexibility of the binding sites examined, especially for receptor tyrosine kinases like EGFR that undergo large domain movements [31]. Nevertheless, the primary objective of this computational analysis—establishing relative binding preferences among targets—remains valid. The consensus ranking of NMDA receptor > CSNK1D ≈ EGFR is preserved across both methods despite differences in absolute scores, supporting the mechanistic interpretation that ketamine acts primarily through NMDA receptor antagonism with indirect effects on downstream targets.

Therefore, the proposed pathway interactions should be interpreted as exploratory and hypothesis-generating rather than definitive mechanistic conclusions.

## 5. Limitation

A further limitation of this study is the use of a basal cell carcinoma-focused qPCR array rather than a colorectal cancer-specific panel. Although the selected genes are involved in common oncogenic pathways, tumor-type-specific differences should be considered.

Another limitation of this study is the use of a single colorectal cancer cell line (HT-29), which may limit the generalizability of the findings. Future studies should include multiple colorectal cancer cell lines with different molecular characteristics, as well as non-malignant colon epithelial cells, to better assess the selectivity and broader applicability of ketamine’s effects.

## 6. Conclusions

In summary, this study provides evidence that ketamine significantly inhibits proliferation and promotes apoptosis in HT-29 colorectal adenocarcinoma cells in vitro. These findings reinforce the emerging notion that anesthetic drugs may influence tumor cell biology beyond their conventional role in perioperative management. By showing that ketamine downregulates key regulators of proliferation and survival pathways, our results suggest that this agent could be further explored as a potential adjuvant in colorectal cancer therapy.

Nevertheless, translation of these in vitro findings to clinical practice requires caution. Factors such as ketamine dose, exposure duration, tumor microenvironment complexity, and the specific enantiomeric form may significantly influence biological outcomes. In particular, emerging evidence suggests that low-dose ketamine, especially S-ketamine, may exert biological effects without inducing full anesthetic states, highlighting the importance of dose optimization in potential clinical applications. Therefore, further in vivo and clinical studies are required to validate these findings and to determine their translational relevance.

Taken together, our results highlight the potential of ketamine as both an anesthetic and a modulator of cancer cell behavior. Further integrative studies combining molecular, cellular, and in vivo approaches are warranted to establish whether this dual role can be leveraged in the design of future perioperative and oncological treatment strategies.

The computational predictions presented here provide a foundation for more rigorous biophysical validation. Future studies should incorporate molecular dynamics simulations (100–200 ns timescales) to assess the stability of predicted binding poses and capture protein flexibility effects [31]. Binding free energy calculations using MM-PBSA or MM-GBSA methods would provide more accurate thermodynamic estimates than docking scores alone. Additionally, experimental validation through surface plasmon resonance (SPR) or isothermal titration calorimetry (ITC) would directly measure ketamine binding affinities for these targets, definitively establishing whether EGFR and CSNK1D are direct or indirect targets. Future studies should validate these findings at the protein level and investigate functional pathway activity to confirm the biological relevance of these transcriptional changes.

Finally, functional assays examining EGFR kinase activity and CSNK1D phosphorylation activity in the presence of ketamine would confirm whether the weak computational binding predictions translate to negligible functional inhibition, as our model suggests [35,48].

## Figures and Tables

**Table 1:** Gene names and expression value in HT-29 cells.

Gene Symbol	Gene Name	FoldChange 2^−ΔΔCq^	Down-Regulation/ Up-Regulation	*p* Value
CSNK1D	Casein kinase 1 delta	0.25	down-regulation	** <0.001
CSNK1E	Casein Kinase 1 Epsilon	1.42	-	>0.05
CASP3	Caspase 3	1.65	-	>0.05
CSNK1G1	Casein kinase 1 gamma 1	1.39	-	>0.05
EGF	Epidermal Growth Factor	0.39	down-regulation	** <0.001
DVL2	Disheveled segment polarity protein 2	1.9	-	>0.05
DVL3	Disheveled segment polarity protein 3	1.87	-	>0.05
FZD2	Frizzled Class Receptor 2	1.56	-	>0.05
FZD3	Frizzled Class Receptor 3	0.81	-	>0.05
FZD4	Frizzled Class Receptor 3	1.17	-	>0.05
GAS1	Growth arrest specific 1	1.62	-	>0.05
KLF4	KLF transcription factor 4	0.59	-	>0.05
PRKACB	Protein Kinase CAMP-Activated Catalytic Subunit Beta	0.88	-	>0.05
PRKACG	Protein Kinase CAMP-Activated Catalytic Subunit Gamma	1.45	-	>0.05
STAT2	Signal transducer and activator of transcription 2	1.72	-	>0.05
BTRC	Beta-Transducin Repeat Containing E3 Ubiquitin Protein Ligase	1.2	-	>0.05
GLI2	GLI family zinc finger 2	0.86	-	>0.05
SUFU	SUFU negative regulator of hedgehog signaling	0.94	-	>0.05
PTCH1	Patched 1	1.43	-	>0.05
WNT1	Wnt family member 1	1.08	-	>0.05
WNT3A	Wnt family member 3A	0.58	-	>0.05
WNT4	Wnt family member 4	0.68	-	>0.05
WNT5A	Wnt family member 5A	1.42	-	>0.05
WNT9A	Wnt family member 9A	1.38	-	>0.05
WNT9B	Wnt family member 9B	1.92	-	>0.05
WNT10A	Wnt family member 10A	0.9	-	>0.05
WNT10B	Wnt family member 10B	0.76	-	>0.05
TCF7	Transcription factor 7	0.41	down-regulation	* |<0.05
ZIC2	Zic family zinc finger 2	1.85		>0.05

* *p* < 0.05; ** *p* < 0.01.

**Table 2:** Comparative molecular docking results for ketamine.

Target	PDB	Resolution (Å)	AutoDock Vina Best (kcal/mol)	Schrödinger Glide Best (kcal/mol)	Consensus Ranking
NMDA Receptor	7EU8	2.90	−6.79	−7.53	1 (Strong)
CSNK1D	4TW9	2.00	−5.90	−5.96	2 (Weak)
EGFR	4HJO	2.40	−6.00	−4.43	3 (Weak)

## Data Availability

The data presented in this study are available on request from the corresponding author.
